# Photochemical Hydroxyl Group Abstraction from *N*-Hydroxypyridine-2(1*H*)-thione Isolated in a Solid Hydrogen Matrix: Photogeneration of 2-Mercaptopyridine

**DOI:** 10.3390/molecules29225472

**Published:** 2024-11-20

**Authors:** Hanna Rostkowska, Maciej J. Nowak, Igor Reva, Leszek Lapinski

**Affiliations:** 1 Institute of Physics, Polish Academy of Sciences, Al. Lotnikow 32/46, 02-668 Warsaw, Poland; rostk@ifpan.edu.pl (H.R.); mjnow@ifpan.edu.pl (M.J.N.); 2 Department of Chemical Engineering, CERES, University of Coimbra, 3030-790 Coimbra, Portugal; reva@eq.uc.pt

**Keywords:** photochemistry, IR spectra, hydroxyl radical, 2-mercaptopyridine-*N*-oxide, cage effect, reactive matrix, pyrithione, omadine, pyridylthiyl radical

## Abstract

Monomers of *N*-hydroxypyridine-2(1*H*)-thione were isolated in low-temperature matrices of solid normal hydrogen (n-H_2_). The matrix-isolated compound was irradiated with UV-B (λ = 305 nm) or UV-A (λ > 360 nm) light. Upon such irradiation, the initial form of *N*-hydroxypyridine-2(1*H*)-thione was completely consumed and converted into photoproducts. 2-Mercaptopyridine and water were identified as the main products of these photochemical transformations. Identification of photoproduced 2-mercaptopyridine is unquestionable. It is based on the identity of two sets of IR bands: (i) the bands observed in the IR spectrum recorded (in a separate experiment) for monomers of 2-mercaptopyridine trapped in an n-H_2_ matrix and (ii) a set of IR bands observed in the spectrum recorded after UV irradiation of *N*-hydroxypyridine-2(1*H*)-thione. It should be emphasized that the UV-induced processes, occurring for *N*-hydroxypyridine-2(1*H*)-thione isolated in an n-H_2_ matrix, lead to products that are significantly different from those generated from the compound trapped in solid Ar or in solid N_2_.

## 1. Introduction

**Scheme 1:**
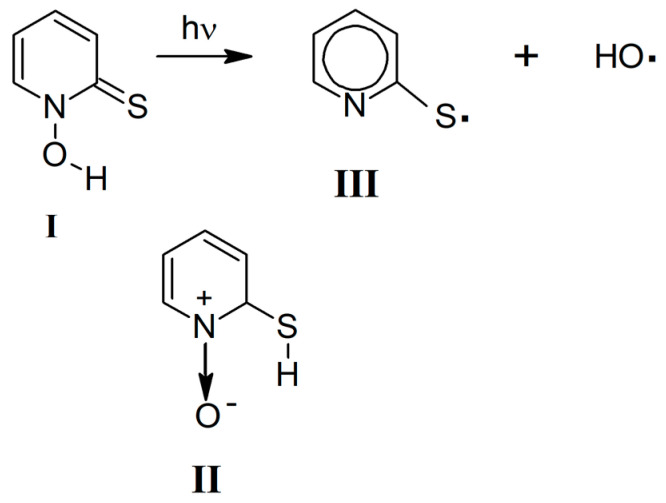
Photochemical N–O bond cleavage and generation of the hydroxyl radical.

*N*-hydroxypyridine-2(1*H*)-thione (IUPAC name 1-hydroxypyridine-2-thione, also known as pyrithione or omadine) has a multitude of applications in the fields of chemistry and biochemistry. The molecule may exist in two tautomeric forms: the thione, *N*-hydroxypyridine-2(1*H*)-thione (**I**), and the thiol, 2-mercaptopyridine *N*-oxide (**II**) (Scheme 1). The latter is the minor tautomer, as evidenced by experimental observations [1]. This is confirmed by theoretical calculations, which showed that in the gas phase, form **I** should be more stable than form **II** by more than 30 kJ mol^−1^ [2,3]. Form **I** was exclusively identified when the molecules were trapped from the gas phase in Ar or N_2_ low-temperature matrices [3]. The compound crystallizes in the most stable thione form [4]. It is known that *N*-hydroxypyridine-2(1*H*)-thione (N-HPT) photochemically decomposes to the hydroxyl •OH and pyridylthiyl (**III**) radicals (Scheme 1) in organic solvents, aqueous media and in a biological environment [5,6]. Therefore, *N*-hydroxypyridine-2(1*H*)-thione may serve as a source of •OH radicals [7,8,9,10].

The hydroxyl radical (•OH) is a significant cause of oxidative damage to proteins and nucleic acids [11,12,13,14]. It reacts at a nearly diffusion-controlled rate with almost any organic biomolecule found in living organisms. The ability of hydroxyl radicals to attack DNA plays an important role in cancer development [15,16,17]. Therefore, *N*-hydroxypyridine-2(1*H*)-thione (**I**) has been extensively utilized as a photochemical source of •OH radicals in investigations on the hydroxyl-radical-induced damage of DNA and other biomolecules. In irradiated aqueous solutions, the N–O bond of the compound dissociates upon excitation with UV-A radiation (λ = 355 nm), resulting in the release of pyridylthiyl **III** and hydroxyl radicals [18,19,20]. Theoretical investigations of the mechanism of UV-induced cleavage of the N–O bond in *N*-hydroxypyridine-2(1*H*)-thione were conducted [2,3]. The results of the calculations indicate that the potential energy surface of the lowest excited singlet state S_1_ is virtually dissociative with respect to detachment of the hydroxyl group. The excited N-HPT molecule undergoes a transition through conical intersections from the potential energy surface (PES) of the ππ* excited state, via the πσ* state, to the ground state of the pyridylthiyl radical.

For *N*-hydroxypyridine-2(1*H*)-thione isolated in Ar and N_2_ matrices, it has been experimentally demonstrated that UV excitation leads to photogeneration of the thioperoxy derivative. This is the result of the recombination of the pyridylthiyl and hydroxyl radicals in the matrix cage [3].

The objective of the current work was to study the photochemical transformations of *N*-hydroxypyridine-2(1*H*)-thione monomers isolated in low-temperature solid hydrogen matrices. Hydrogen matrices have different properties than noble gas matrices. The large amplitude of the zero point lattice vibration of solid hydrogen (which is due to the small mass of the H_2_ molecules and the relatively weak intermolecular forces) [21] gives an effect of softness to the H_2_ matrix. In a soft environment of the hydrogen matrix, the photochemical behavior of N-HPT may diverge significantly from that of *N*-hydroxypyridine-2(1*H*)-thione isolated in rigid argon or nitrogen matrices, which has been previously investigated [3,22,23,24]. In a soft solid hydrogen environment, the photogenerated pyridylthiyl **III** and hydroxyl radicals do not necessarily need to recombine, as was observed in an Ar and N_2_ matrix. It is possible that they may separate and react with the surrounding H_2_ molecules [25]. The aim of the experiments carried out in the present work was to verify whether these expectations would be fulfilled and to identify the products of the photoreactions of *N*-hydroxypyridine-2(1*H*)-thione molecules isolated in solid hydrogen matrices.

## 2. Results

### 2.1. Structure and Infrared Spectrum of N-Hydroxypyridine-2(1H)-thione Monomers Isolated in Low-Temperature n-H_2_ Matrix

**Figure 1:**
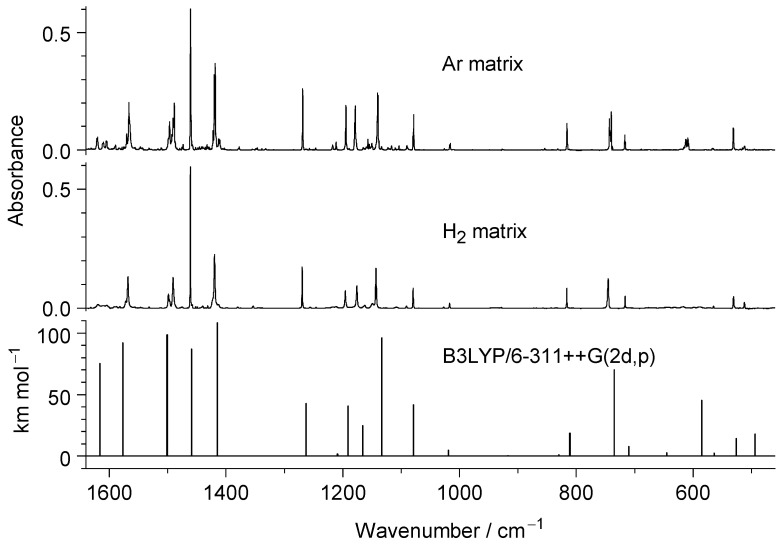
Infrared spectra of *N*-hydroxypyridine-2(1*H*)-thione monomers isolated in an Ar matrix (**upper panel**) and in an n-H_2_ matrix (**middle panel**), compared with the theoretical infrared spectrum (**bottom panel**) calculated at the DFT(B3LYP)/6-311++G(2d,p) level for the *N*-hydroxy-thione isomer **I** of the compound. The theoretical wavenumbers, calculated within the harmonic approximation, were scaled by 0.98.

The title compound of this paper is often referred to as 2-mercaptopyridine-*N*-oxide (structure II in Scheme 1). However, the energy of the *N*-oxide form **II** of *N*-hydroxypyridine-2(1*H*)-thione is significantly higher (by more than 30 kJ mol^−1^) [3] than the energy of *N*-hydroxy tautomer **I**. Consequently, only the *N*-hydroxy form **I** should be populated in the gas phase [26] and trapped in low-temperature Ar or n-H_2_ matrices. The infrared spectra of *N*-hydroxypyridine-2(1*H*)-thione monomers isolated in Ar and in n-H_2_ matrices are presented in Figure 1. The spectra of the compound isolated in Ar and n-H_2_ matrices are very similar to each other. The apparent exception concerns the bands at 613 and 609 cm^−1^ (Ar), whose counterpart(s) appear to be quite weak in the spectrum of the compound isolated in an n-H_2_ matrix. Such low relative peak intensities of the bands due to the out-of-plane τOH vibrations are typical for the infrared spectra of compounds isolated in solid hydrogen matrices [22]. The experimental spectra of *N*-hydroxypyridine-2(1*H*)-thione isolated in Ar and n-H_2_ matrices are well reproduced by the theoretical spectrum calculated for the *N*-hydroxy tautomer **I**. This confirms that the *N*-hydroxy form **I** is adopted by monomers of *N*-hydroxypyridine-2(1*H*)-thione isolated in low-temperature Ar and n-H_2_ matrices.

### 2.2. UV Irradiation of N-Hydroxypyridine-2(1H)-thione Isolated in a Low-Temperature n-H_2_ Matrix

**Figure 2:**
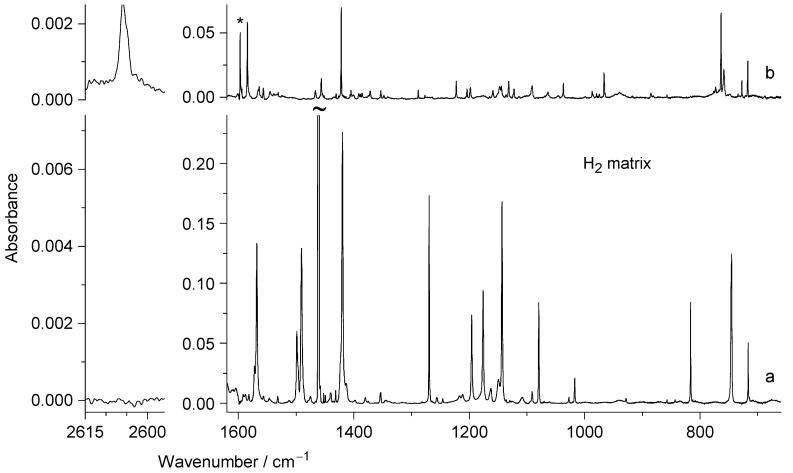
Fragments of the infrared spectra of *N*-hydroxypyridine-2(1*H*)-thione monomers isolated in an n-H_2_ matrix: (**a**) recorded before any irradiation; (**b**) recorded after 15 min of UV (λ = 305 nm) irradiation of the matrix. Asterisk indicates the band due to photoproduced H_2_O.

Monomers of *N*-hydroxypyridine-2(1*H*)-thione isolated in an n-H_2_ matrix were irradiated with UV (λ = 305 nm) light. Upon 15 min of such irradiation, the initial form of the compound was entirely depleted (see Figure 2). The initial IR spectrum disappeared completely, whereas the new spectrum of the photoproducts emerged. In the new IR spectrum, the band at 1598 cm^−1^ indicates that H_2_O is one of the photoproduced species.

**Scheme 2:**
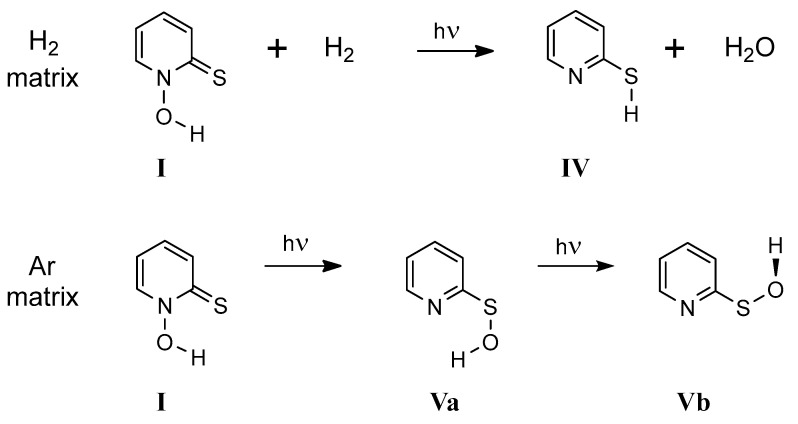
Photochemical transformations of *N*-hydroxypyridine-2(1*H*)-thione monomers isolated in n-H_2_ and Ar matrices.

**Figure 3:**
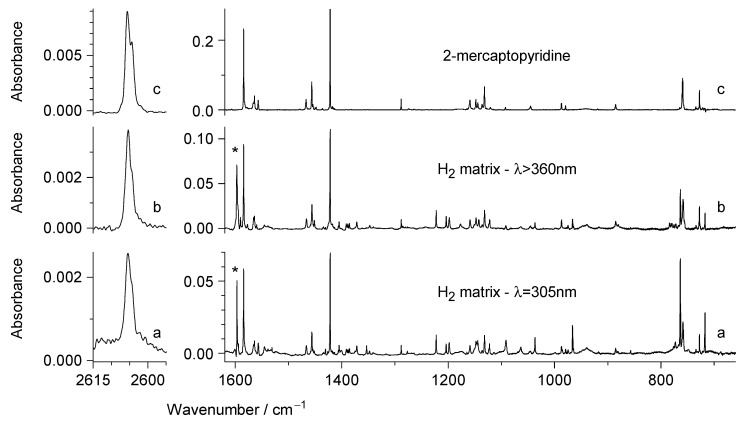
Fragments of the infrared spectra of (**a**) *N*-hydroxypyridine-2(1*H*)-thione monomers isolated in an n-H_2_ matrix and irradiated for 15 min with UV (λ = 305 nm) light; (**b**) *N*-hydroxypyridine-2(1*H*)-thione monomers isolated in an n-H_2_ matrix and irradiated for 180 min with UV (λ > 360 nm) light; (**c**) 2-thiopyridine monomers trapped (in a separate experiment) from the gas phase in an n-H_2_ matrix. Asterisks indicate the bands due to photoproduced H_2_O.

Another new band, found at 2606 cm^−1^ in the spectrum recorded after UV (λ = 305 nm) irradiation of the matrix, shows that there should be a mercapto (-S–H) group in the structure of one of the photoproducts. One of the candidates for such a structure is 2-mercaptopyridine (**IV**, see Scheme 2), the thiol form of 2-thiopyridine. In a separate, dedicated experiment, monomers of 2-thiopyridine were trapped in an n-H_2_ matrix. The infrared spectrum of this matrix is presented in Figure 3c. All the IR bands present in this spectrum have their counterparts (found at exactly the same wavenumbers) in the spectrum of the photoproducts generated upon UV (λ = 305 nm) irradiation of *N*-hydroxypyridine-2(1*H*)-thione isolated in an n-H_2_ matrix (compare traces a and c in Figure 3). This provides a very strong proof for the hypothesis that the main products generated upon UV (λ = 305 nm) irradiation of *N*-hydroxypyridine-2(1*H*)-thione isolated in an n-H_2_ matrix are 2-mercaptopyridine **IV** and H_2_O (see Scheme 2). In another experiment, we also checked that upon longer-wavelength UV (λ > 360 nm) irradiation of monomeric *N*-hydroxypyridine-2(1*H*)-thione, the same two species are generated (as main photoproducts) in an n-H_2_ matrix (see Figure 3 traces a and b).

**Figure 4:**
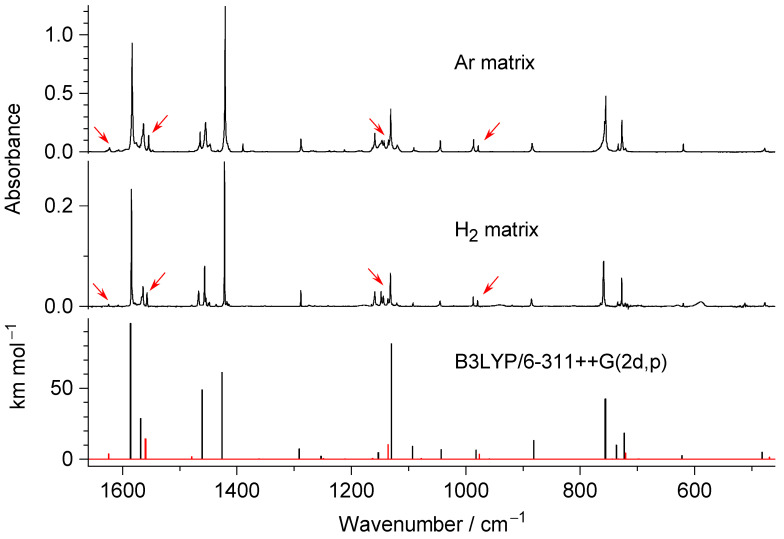
Infrared spectra of 2-mercaptopyridine monomers isolated in an Ar matrix (**upper panel**) and in an n-H_2_ matrix (**middle panel**), compared with the theoretical spectrum (**bottom panel**) calculated at the DFT(B3LYP)/6-311++G(2d,p) level for the thiol (black) and thione (red) tautomer of the compound. The intensities of the theoretically calculated IR bands due to the thione tautomer of the compound were multiplied by 0.05. The theoretical wavenumbers, calculated within the harmonic approximation, were scaled by 0.98. The experimental bands due to the thione form of 2-thiopyridine are marked with arrows.

2-Thiopyridine is a compound that can adopt the thione or the thiol tautomeric form. The thiol form **IV** is more stable (by ca. 10 kJ mol^−1^) than the thione tautomer [27,28]. That is why the thiol form **IV** of the compound (2-mercaptopyridine) should be nearly exclusively populated in the gas phase and trapped in low-temperature Ar and n-H_2_ matrices. The infrared spectra of monomers of 2-thiopyridine isolated in Ar and n-H_2_ matrices are presented in Figure 4. The experimental spectra of the matrix-isolated compound are very well reproduced by the theoretical spectrum calculated for the thiol tautomer. This shows that the spectrum presented in trace b of Figure 3 is dominated by the spectrum of the thiol tautomer of 2-thiopyridine. Hence, this form of the compound (2-mercaptopyridine) **IV** is one of the main photoproducts generated upon UV (λ = 305 nm) irradiation of *N*-hydroxypyridine-2(1*H*)-thione isolated in an n-H_2_ matrix (Scheme 2).

In a low-temperature matrix obtained by co-deposition of 2-thiopyridine vapor and large excess of matrix gas (Ar or n-H_2_), the population of the thione form of the compound is low [27,28]. Nevertheless, because of high absolute intensities of IR bands due to the thione tautomer, a series of such bands [3400, 1554, 978 cm^−1^ (Ar); 3399, 1557, 979 cm^−1^ (n-H_2_)] can be found in the spectrum of a matrix containing monomers of 2-thiopyridine (see Figure 4). Interestingly, the series of bands at 3399, 1557, 979 cm^−1^ (n-H_2_) was also found in the spectrum of *N*-hydroxypyridine-2(1*H*)-thione isolated in an n-H_2_ matrix and irradiated with UV (λ = 305 nm) light. This demonstrates that a small amount of the thione form of 2-thiopyridine is also generated (together with a large amount of the thiol form of 2-thiopyridine) upon UV irradiation of *N*-hydroxypyridine-2(1*H*)-thione isolated in an n-H_2_ matrix.

Alongside the main photoproducts (2-mercaptopyridine **IV** and water), some other unidentified species were photogenerated when *N*-hydroxypyridine-2(1*H*)-thione isolated in an n-H_2_ matrix was irradiated with UV light. The IR bands observed at 1639, 1633, 1222, 1204, 1198, 1037, 966, 764, 717 cm^−1^ are the spectral signatures of these photoproducts. One of these products, which is characterized by the bands 1037, 966, 764, 717 cm^−1^, is generated in smaller amounts upon longer-wavelength irradiation (λ > 360 nm) of *N*-hydroxypyridine-2(1*H*)-thione isolated in an n-H_2_ matrix and in larger amounts when UV (λ = 305 nm) light is used for irradiation (Figure 3). According to this observation, at least two different minor photoproducts were generated under such conditions.

**Figure 5:**
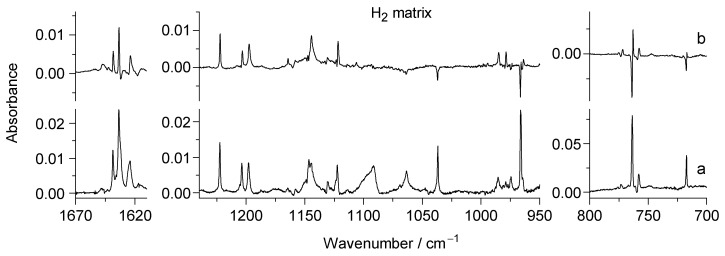
Fragments of (**a**) extracted infrared spectrum of minor photoproducts generated upon UV (λ = 305 nm) irradiation of *N*-hydroxypyridine-2(1*H*)-thione monomers isolated in an n-H_2_ matrix, juxtaposed with (**b**) the difference spectrum obtained by subtracting the spectrum of UV- (λ = 305 nm) irradiated monomers of *N*-hydroxypyridine-2(1*H*)-thione monomers isolated in an n-H_2_ matrix from the spectrum registered after subsequent 24 h of keeping the irradiated matrix at 3.5 K in the dark.

The remark above is consistent with the results of a spontaneous process that occurred when the UV-irradiated n-H_2_ matrix, originally containing the products photogenerated from *N*-hydroxypyridine-2(1*H*)-thione monomers, was kept at 3.5 K and in the dark for 24 h (Figure 5). During this time, some bands belonging to the spectrum of the photoproducts were growing, while the others were decreasing (Figure 5b). The increasing bands observed at 1639, 1633, 1222, 1198, 1144, and 1122 cm^−1^ evidently originated from the spectrum of one minor photoproduct, whereas another set of diminishing bands observed at 1037, 966, 764, and 717 cm^−1^ clearly indicates the presence of the second minor photoproduct, generated upon UV (λ = 305 nm) irradiation. The carriers of these bands currently remain unknown.

### 2.3. Comparison of Photoproducts Generated upon UV Irradiation of N-Hydroxypyridine-2(1H)-thione Isolated in n-H_2_ Matrices with Those Photoproduced from the Compound Isolated in Ar or N_2_ Matrices

**Figure 6:**
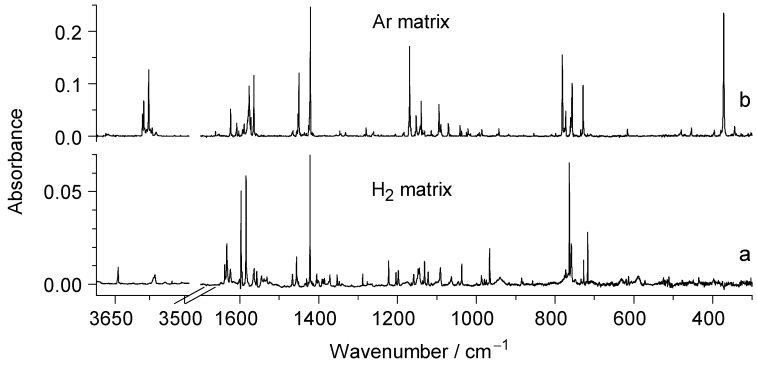
Infrared spectra of photoproducts generated from *N*-hydroxypyridine-2(1*H*)-thione monomers isolated in (**a**) an n-H_2_ matrix, recorded after 15 min of UV (λ = 305 nm) irradiation; (**b**) an Ar matrix, recorded after 15 min of UV (λ = 305 nm) irradiation.

The final products resulting from UV excitation of *N*-hydroxypyridine-2(1*H*)-thione isolated in n-H_2_ matrices are very different from those resulting from UV excitation of monomers of the compound isolated in Ar or N_2_ matrices. The IR spectrum recorded after total photochemical transformation of the initial form **I** of *N*-hydroxypyridine-2(1*H*)-thione isolated in n-H_2_ matrices into the photoproducts (Figure 6) does not resemble the spectrum of the photoproducts generated from monomers of the compound isolated in Ar or N_2_ matrices. 

Different IR spectra reflect the fact that the products generated in the photochemical transformations of *N*-hydroxypyridine-2(1*H*)-thione isolated in an n-H_2_ matrix are very different from those photoproduced from the compound isolated in Ar or N_2_ matrices. As was demonstrated in the previous work [3], two conformers of a thioperoxy derivative (**Va** and **Vb** in Scheme 2) were photogenerated upon UV excitation of N-HPT isolated in Ar or N_2_ matrices. No traces of such products were detected for *N*-hydroxypyridine-2(1*H*)-thione isolated in solid hydrogen and subjected to UV irradiation. In the IR spectra of *N*-hydroxypyridine-2(1*H*)-thione monomers isolated in n-H_2_ matrices and irradiated with UV (λ = 305 nm or λ > 360 nm) light, no spectral indications of **Va** or **Vb** products could be found. Neither a counterpart of a very characteristic band (at ca. 3577 cm^−1^) due to the νOH vibration in form **Vb**, nor a counterpart of the band at 782 cm^−1^ (characteristic of form **Va**), could be found in the IR spectra of *N*-hydroxypyridine-2(1*H*)-thione monomers isolated in n-H_2_ matrices and irradiated with UV (λ = 305 nm or λ > 360 nm) light. And vice versa, when *N*-hydroxypyridine-2(1*H*)-thione was isolated in Ar or N_2_ matrices and excited with UV light, no spectral indications of photoproduced 2-mercaptopyridine could be detected in the infrared spectra recorded after the UV irradiation.

## 3. Discussion

In the current work, we have studied the effects of UV-B (λ = 305 nm) or UV-A (λ > 360 nm) excitation of *N*-hydroxypyridine-2(1*H*)-thione molecules isolated in low-temperature normal hydrogen (n-H_2_) matrices. Two main products of these phototransformations (2-mercaptopyridine **IV** and H_2_O) were reliably identified by comparison of their infrared spectra with the spectra of these species isolated (in separate experiments) in low-temperature n-H_2_ matrices. Such products were not photogenerated for the compound isolated in solid Ar or in solid N_2_. Instead, thioperoxy derivatives **Va** and **Vb** were photoproduced when *N*-hydroxypyridine-2(1*H*)-thione monomers isolated in Ar or N_2_ matrices were excited with UV light.

It is easy to imagine how these forms (**Va** and **Vb**) may be photoproduced. In the first step following the UV excitation, the N–O bond is broken and two radicals (hydroxyl and pyridylthiyl **III**, Scheme 1) are photogenerated. Photochemical cleavage of the N–O bond is quite a common phenomenon [29,30,31] and it seems to follow the mechanism involving dissociation on the surface of the repulsive state with the πσ* character [2,3]. In the second step, the hydroxyl and pyridylthiyl radicals recombine and a new S–O bond is formed. Recombination of the radicals is enforced by the strong caging effect exhibited by the Ar and N_2_ matrices. Low-temperature argon matrices (such as krypton, xenon and nitrogen matrices) are known for a very pronounced cage effect [32]. 

The matrices of solid hydrogen are much softer. In such matrices, the cage effect is significantly less pronounced [21,33,34]. Hence, in soft n-H_2_ matrices, the recombination of the photoproduced fragments (hydroxyl and pyridylthiyl radicals) is not enforced. After the dissociation of the N–O bond, the generated radicals can move apart from each other. Moreover, in n-H_2_ matrices, both pyridylthiyl and hydroxyl radicals can react with H_2_ molecules to give 2-mercaptopyridine and H_2_O products, respectively (Scheme 2). Reaction of hydroxyl radicals with hydrogen molecules has been reported previously [31]. In a very detailed study on UV-induced dissociation of HONO molecules isolated in solid *para*-hydrogen, it was demonstrated that photoproduced hydroxyl radicals react with the solid hydrogen environment to form the H_2_O product. Reactions with H_2_ molecules of the solid hydrogen environment were also observed for methyl radicals [35,36], as well as for ·NH_2_ and :NH radicals [37].

## 4. Materials and Methods

The sample of *N*-hydroxypyridine-2(1*H*)-thione used in the current study was a commercial product purchased from Sigma-Aldrich (St. Louis, MO, USA). The solid compound was placed in a glass tube connected by a regulating valve to the inner part of the cryostat shroud. To deposit a low-temperature matrix, vapor of *N*-hydroxypyridine-2(1*H*)-thione was frozen together with a large excess of matrix gas (argon or normal hydrogen) onto a CsI substrate cooled to 3.5 K by a closed-cycle Sumitomo SRDK-408D2 (Osaka, Japan) helium cryostat. Normal hydrogen (n-H_2_), which is a 3:1 mixture of *ortho*- and *para*-hydrogen, was used in these experiments. Matrices containing 2-thiopyridine monomers were prepared in a slightly different way. The solid sample of this compound (Sigma-Aldrich) was placed in a miniature glass oven located inside the vacuum shroud of the cryostat. The vapor of 2-thiopyridine, coming out of the electrically heated oven, was deposited on the cold CsI window simultaneously with Ar or n-H_2_ matrix gas. The mid-infrared spectra were collected with 0.5 cm^−1^ resolution using a Thermo Nicolet iS50R FTIR spectrometer (Waltham, MA, USA) equipped with a KBr beam splitter and a DTGS detector with a KBr window. The spectra in the lower-wavenumber 700–300 cm^−1^ range were recorded using the same spectrometer but equipped with a “solid substrate” beam splitter and a DTGS detector with a polyethylene window. Matrix-isolated monomers of *N*-hydroxypyridine-2(1*H*)-thione were irradiated with quasi-monochromatic UV light (λ_max_ = 305 nm, FWHM = 15 nm, optical power = 100 mW) emitted by a 6060 LG Innotek diode. In some experiments, matrices were irradiated with UV (λ > 360 nm) light emitted by an HBO 200 high-pressure mercury lamp equipped with a WG 360 Schott longpass filter and water filter.

The geometries of the molecules considered in the current work were optimized at the DFT(B3LYP) level [38,39,40]. At the optimized geometries, infrared spectra were calculated within the harmonic approximation. The standard 6-311++G(2d,p) basis set was applied in these computations. The calculations were performed using the Gaussian 09 program suite [41]. The computed wavenumbers higher than 2000 cm^−1^ were scaled with a factor of 0.95, whereas the wavenumbers lower than 2000 cm^−1^ were scaled with a factor of 0.98.

## Data Availability

The data presented in this study are available upon request from the corresponding author.
